# Color Changes of UHT Milk During Storage

**DOI:** 10.3390/s8095961

**Published:** 2008-09-25

**Authors:** Jovanka V. Popov-Raljić, Nada S. Lakić, Jovanka G. Laličić-Petronijević, Miroljub B. Barać, Višnja M. Sikimić

**Affiliations:** 1 University of Belgrade, Faculty of Agriculture, Belgrade, Nemanjina 6, Serbia; E-Mails: nlakic@agrifaculty.bg.ac.yu (N.S.L.); jovankal@agrifaculty.bg.ac.yu (J.G.L.P.); baracm@agrifaculty.bg.ac.yu (M.B.B.); 2 High Technical School of Vocational Studies, Pozarevac, Nemanjina 2, Serbia; E-Mail: visnja.sikimic@gmail.com (V.M.S.)

**Keywords:** UHT milk, color measurement, milk fat content, storage

## Abstract

In this study measurements of color parameters of UHT milk were performed, by using a MOM-color 100 photoelectric tristimulus colorimeter. Colors of UHT milk samples containing 3.2% and 1.6% milk fat, processed under industrial conditions, packed in polyethylene terephtalate (PET) based packages, and stored for 0, 15, 30, 45, 60 and 90 days at ambient temperature (20±5°C) were examined. Results are shown in four different systems that define measurement of color parameters expressed in: CIE, CIE L*a*b*, Hünter and ANLAB – Adams Nickerson systems. Average value of mean reflectance of UHT milk determined in CIE system statistically is highly significantly changed, (p < 0.01) depending on duration of storaging, percentage of milk fat, as well as on the interaction of the mentioned factors. For the UHT milk with 1.6% milk fat statistically significant (p < 0.05) decrease of psychometric chroma b* occurs after 60 days, and for milk with 3.2% milk fat established on 45^th^ day of storage.

## 1. Introduction

One of the principal goals of milk preservation methods by its short time treatment at increased temperatures is to obtain the desired degree of destruction of microorganisms and of inactivation of enzymes, with, at the same time, introducing the least possible undesired changes of physico-chemical and sensory properties, as well as, what is even more important, preservation of its nutritional value.

From the point of view of sensorial quality characteristics, appearance – color, viscosity, odor and taste – is important for consumers. Appearance – color of the UHT milk samples having different percentual fat contents – can be defined in scope of visual (sensorial) impressions, as well the scopes of different chemical, microanalytical and instrumental methods [1-12].

The color of the UHT milk, i.e. its intensity, basically represents reflection of physico-chemical changes in the product. Gaucher *et al.* [13] examined the effects of storage of partially defatted UHT milk on its particular physico-chemical characteristics. UHT milk was stored up to 6 months at different temperatures (4, 20 and 40°C). These authors concluded that during storage complex physico-chemical changes of milk occur, and that an increase of storage temperature essentially affects the rate and degree of individual changes. Thus, the acidification (increase of acidity) can be explained and with the psychometric chroma (b*) increase, which Kneifel *et al.* [14] define as an indicator that determines problems (defects, damages) ocurring during storage, especially with respect to nonenzymatic milk spoilage. These reactions are known as Maillard's reactions, which start with binding of aldehyde group of lactose with ε-amino group of the lysyl – residues (amino-acid radical, or residue of amino-acid lysine) from different milk proteins [15]. These reactions consist of a series of changes whose consequence is the formation of brown-colored pigments, such as pyralysins and melanoidins, polymers such as lactulose-lysine or fructose-lysine, as well as low-molecular weight acids.

Large number of food products, including milk and milk products, are susceptible to oxidation. Milk products are especially sensitive to light-induced oxidation because of the presence of riboflavin (vitamin B_2_), which is very sensitive on light and it can absorb visible and UV light, converting this energy into highly reactive forms of oxygen [16]. On the other hand, this can induce a whole series of oxidative reactions, which, as consequence, cause significant losses of valuable nutrients such as vitamins (including vitamins A, B_2_, C, D and E), and amino-acids, as well as oxidation of fat, discoloration and creation of undesirable odors [17].

To avoid the aforementioned changes during storage of the UHT milk, the choice of the packaging material plays a very important role. In this regard a great number of factors can influence the degradation kinetics of riboflavin, such as distance to the light source, intensity of radiation, wavelength, duration of exposure to light and temperature, thermal processing and milk homogenization [18, 19, 6].

The objectives of these investigations were instrumental measurement of the UHT milk color parameters of milk samples with 3.2% and 1.6% milk fat during prolonged storage (up to 90 days). Measurements of color parameters are relatively easy and fast by using instrumental methods. On the other hand, changes of color parameters indicate other, deeper physico-chemical sensorial and structural changes of products, which were beyond the scope of our present investigations.

## 3. Experimental Section

Industrially processed UHT milk, packed in polyethylene-based (PET) packaging, was used in this study of the instrumental determination of the color. Samples were taken from the central milk processing plant of the city of Novi Sad (Serbia) during an average working day after application of standard pretreatment and the UHT processing technology (137 – 142°C, 2 – 3 seconds).

The first group of 30 samples were UHT milk samples containing 3.2% milk fat, and the second group were 30 samples of UHT milk with 1.6% milk fat. The declared compositions of these samples were: milk fat 3.2/1.6 g/100 g of products, proteins 3.2/3.18 g/100 g, carbohydrates 4.7/4.85 g/100 g, and energetic value of 260/199 kJ/100 g, respectively. Five samples of each group were analyzed after the specified storage periods, according to the experimental design. Samples with 3.2% and 1.6% milk fat were chosen because these types of milk prevail on Serbian market, being preferred by consumers.

The color parameters of the UHT milk samples were measured with a MOM-color 100 photoelectric tristimulus colorimeter, immediately after processing (day 0), and after storage for 15, 30, 45, 60 and 90 days at room temperature (20 ±5°C), simulating conditions prevailing during the sale of milk from supermarket shelves. The given storage temperature was chosen, because in Serbia in small markets almost exclusively, and in large supermarkets very often, UHT milk prepared for retail trade is kept on open shelves, at room temperatures. Results are expressed in four systems of defining of color, and namely: CIE, CIE L a*b, ANLAB and Hünter's systems.

Apparatus was calibrated with the standard white "observer", which is characterized by the following tristimulus values: *x_1_* = 63.21; *x_2_* = 15.81; *y* = 81.28 and *z* = 95.01, and can be perceived as the “standard eye” with filters for standard colors (red, green and blue) [20]. The sample of the examined milk is placed in the provided space in the glass cell (0.5 cm high and 2.5 cm diameter) and corresponding tristimulus values were read directly on the apparatus MOM color. Trichromatic coefficients *X* and *Y* are calculated from the following equations:

(1) $$X=\frac{x}{x+y+z}\;\text{and}\;Y=\frac{y}{x+y+z}$$

with: *x* = *x_1_* + *x_2_*

**Figure 1.**
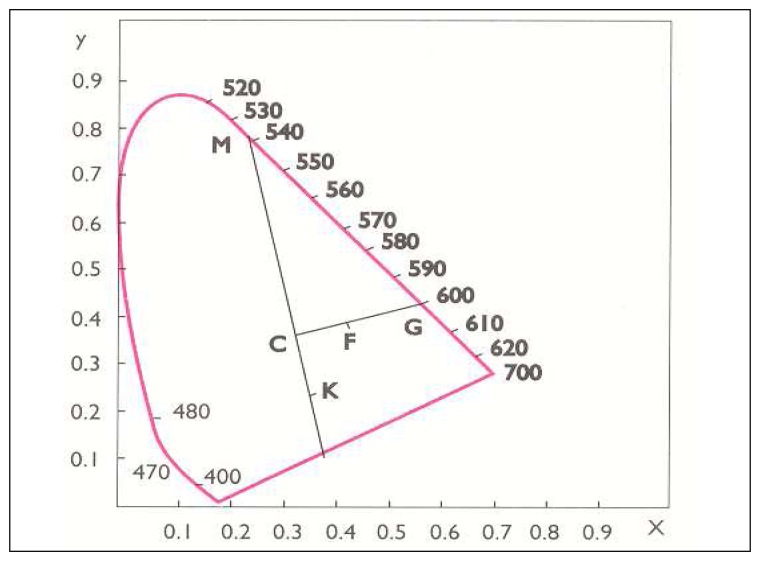
Determination of dominant wavelength and purity of color by CIE system.

Calculated trichromatic coefficients are used for calculation of the color purity expressed in percents, and simultaneously reading of the dominant wavelength, on the base of the chromaticity diagram [21]. Dominant wavelength (λ) is determined on the basis of the calculated trichromatic coefficients *X* and *Y,*, which are introduced into the chromaticity diagram as point F, which is to be jointed with the point C, and extended to the intersection with the spectral curve. Point of intersection (point G) represents the dominant wavelength (Figure 1).

Color purity is expressed in per cents and it is to be calculated on the basis of the following relation:

(2) $$\text{color purity}\;(\%)\;P=\frac{\bar{C}\bar{F}}{\bar{F}\bar{G}}\cdot 100$$

where *C̅F̅* represents the distance of points C and F, and *F̅G̅* the distance of points F and G.

In the CIE system, average reflectance or brilliance is determined on the basis of the Y (%) – value which is read directly at the MOM – color.

In the CIELab system, color quality parameters are expressed on the basis of the following equations:

(3) $$\text{psychometric lightness}\;L*=116\cdot {\left(\frac{Y}{Y_{0}}\right)}^{\frac{1}{3}}-16$$

(4) $$\text{psychometric tone}\;a*=500\cdot \left[{\left(\frac{X}{X_{0}}\right)}^{\frac{1}{3}}-{\left(\frac{Y}{Y_{0}}\right)}^{\frac{1}{3}}\right]$$

(5) $$\text{psychometric chroma}\;b*=200\cdot \left[{\left(\frac{Y}{Y_{0}}\right)}^{\frac{1}{3}}-{\left(\frac{Z}{Z_{0}}\right)}^{\frac{1}{3}}\right]$$

a* - psychometric tone [participation of red (+) and green (−) colors of components];

b* - psychometric chroma [participation of yellow (+) and blue (−) colors of components].

Color difference with respect to the standard white, according to Robertson [22], is defined as:

(7) $$\Delta H_{ab}^{*}=C^{*}\cdot \Delta h*.\frac{\pi }{180}$$

According to Hünter, the mentioned values are calculated on the base of the following equations:

(8) $$\text{psychometric lightness}:L_{\text{Hu}}=100\sqrt{\frac{Y}{Y_{n}}}$$

(9) $$\text{psychometric tone}:a_{\text{Hu}}=K_{a}\left[\frac{\frac{X}{X_{n}}-\frac{Z}{Z_{n}}}{\sqrt{\frac{Y}{Y_{n}}}}\right]$$

(10) $$\text{psychometric Chroma}:b_{\text{Hu}}=K_{b}\left[\frac{\frac{Y}{Y_{n}}-\frac{Z}{Z_{n}}}{\sqrt{\frac{Y}{Y_{n}}}}\right]$$

where *X, Y, Z* represent CIE tristimulus values,

*X_n_, Y_n_, Z_n_* are tristimulus values taken from tables connected with the light source
*K_a_, K_b_* are coefficients of chromaticity for the light source, and
*Y_n_* = 100.00 – for each occasion.

For determination of the degree of difference of color between sample and the standard white, there is possibility of calculation of Δ*E_Hü_*-values:

(11) $$\Delta E_{Hu}=\sqrt{{\left(\Delta L_{\text{Hu}}\right)}^{2}+{\left(\Delta a_{\text{Hu}}\right)}^{2}+{\left(\Delta b_{\text{Hu}}\right)}^{2}}$$

### Statistical evaluation of the obtained results

Information about the results of investigations are given as basic parameters of the descriptive statistics: arithmetic mean, standard deviation and variation coefficient. Equality of variances of the analyzed treatments was examinated with the Levene test and, in accordance with the obtained results, experimental data are processed with corresponding model of analyze of variance (MANOVA) and with the LSD – test. Statistical analysis of experimental data is performed with the STATISTICA v.6 – package.

## 2. Results and Discussion

Results of instrumental determination of color quality parameters of UHT milk containing 3.2% and 1.6% milk fat on photoelectrical tristimulus colorimeter MOM-color 100, are shown in tabular form (Tables 1 and 2).

**Figure 2.**
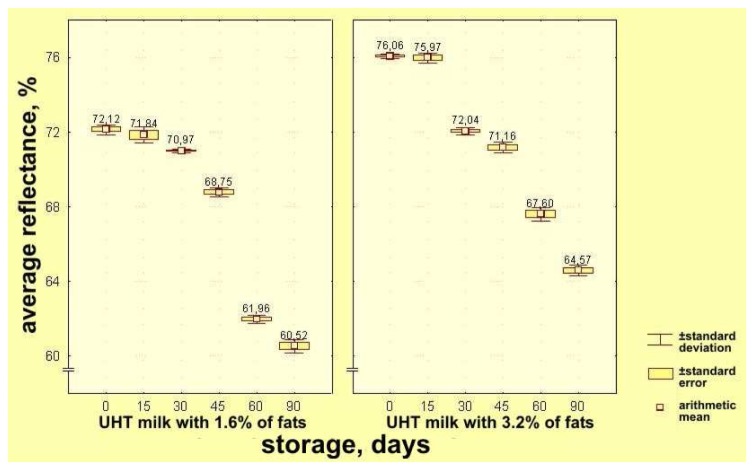
Effects of storage time on changes of average reflectance of UHT milk samples with 1.6 and 3.2% milk fat represented in the CIE system.

On the basis of the results obtained for UHT milk samples with 3.2% milk fat, displayed in the CIE system, average values of the mean reflectance immediately after processing were the highest, and were-: y = 76.060%, with standard deviation value of S = 0.139 and coefficient of variation of C_v_ = 0.183%. During storage up to 15 days, values of average reflectance decreased insignificantly, if compared with their initial values, y = 75.970%, with S = 0.274 and C_v_ = 0.361%. During the prolonged storage samples of UHT milk with 3.2% fat, after 30, 45, 60 and 90 days, showed further insignificant decreases: after 90 days y = 64.570%, with S = 0.296 and C_v_ = 0.458% (Table 1, Figure 2).

As it can be seen from the Table 2, the highest values of average reflectance or color brilliance corresponded to UHT milk samples with 1.6% milk fat immediately after being processed (y = 72.120%, with S = 0.265 and C_v_=0.367%); they gradually decrease insignificantly and after 15 days have values y = 71.840%, with S = 0.442 and C_v_ = 0.616%). During further storage of samples of UHT milk with 1.6% milk fat the decrease of their average reflectance continues and after 90 days, values of y is y = 60.520% with S = 0.361 and C_v_ = 0.596% were obtained.

According to the results obtained with MANOVA, average values of average reflectance, i.e. of brilliance of UHT milk, are subjected to statistically significant (p < 0.01) changes under the influence of the analyzed factors: *storage time* (F = 1752.216 and p = 0.000) and *milk fat content* (F = 1428.497 and p = 0.000), as well as with interactions of the mentioned factors (F = 47.590 and p = 0.000).

Average value of average reflectance, i.e. of brilliance – lightness of color of UHT milk samples, without respect to their milk fat contents, were not significantly changed statistically (p > 0.05) in the period between days 0 and 15, but, in periods of 30, 45, 60 and 90 days of storage, changes (average) of the analyzed samples for their brilliance were statistically very significant (p < 0.01).

When UHT milk samples with 1.6% and 3.2% milk fat were analyzed separately, on the basis of LSD test results it is possible to say that, for UHT milk samples with 3.2% milk fat (average) brilliance or color lightness, change statistically very significantly (p < 0.01) during the whole period of storage (0, 15, 30, 45, 60 and 90 days). Nevertheless, for samples of UHT milk with 1.6% milk fat, changes of (average) brilliance or color lightness were not statistically significant (p > 0.05) during interval between days 0 and 15, but for all other periods (30, 45, 60 and 90 days) changes were statistically very significant (p < 0.01).

Generally, if compared the color brilliance values of UHT milk samples with 3.2% and 1.6% milk fat, during their storage up to 90 days at 20 ± 5°C, the samples with 3.2% fat had significantly lighter color, i.e. their surfaces were significantly lighter, if compared with the ones with 1.6% milk fat.

**Figure 3.**
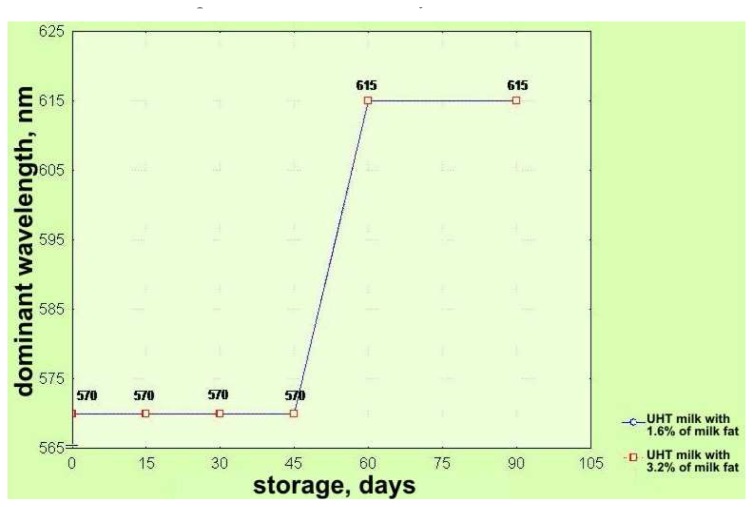
Effects of storage time on changes of color nuances of UHT milk samples with 1.6% and 3.2% milk fat represented in the CIE system.

**Figure 4.**
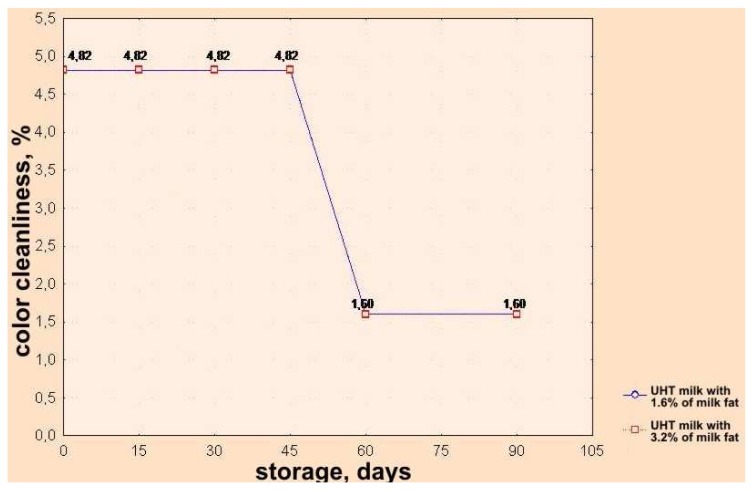
Effects of storage time on changes of color purity of UHT milk samples with 1.6% and 3.2% milk fat represented in the CIE system.

Based on the chromaticity diagram, as well as on the base of calculated trichromatic coefficients in CIE system (Figure 1) [21], values for dominant wavelength λ (nm) (color nuance) and degree of color purity, i.e. of saturation of color were found. Both groups of analyzed UHT milk samples (3.2 % milk fat and 1.6% milk fat) stored up 60 days have the same level of purity (saturation i.e. color nuance); after further storage color changes (darkening) and a decrease of saturation occur. (Figures 3 and 4).

To obtain more precise definition of changes of parameters of color quality of UHT milk samples having different milk fat contents, results of instrumental determination are expressed in CIE L*a*b*, ANLAB and Hünter's systems, based on psychometric lightness – L*, L, and L_Hu_; psychometric tone – a*, A, a_Hu_ – participation of red (+) and green (−) components of milk color; psychometric chroma – b*, B, b_Hu_ – participation of yellow (+) and blue (−) components of milk color, as well as ΔH_ab_, ΔE_AN_ i.e. ΔE_Hu_ values – differences of colors in comparison with the standard white (Tables 1 and 2).

Similarly to the decreasing tendency of average reflectance – color brilliance of UHT milk samples with 3.2% and 1.6% milk fat, it can be observed that values for psychometric lightness in each of the three systems, decrease during storage. Immediately after processing of UHT milk with 3.2% milk fat, values are higher, so that in the CIE L*a*b* system they reach L* = 89.880 with S = 0.339 and C_v_ = 0.377%, in ANLAB system L = 82.500 with S = 0.263 and C_v_ = 0.318%, and in Hünter's system L_Hu_ = 87.210 with S = 0.282 and C_v_ = 0.323%. After 90 days of storage these values decreased to L* = 77.150 with S = 0.394 and C_v_ = 0.510%, i.e. L = 77.150 with S = 0.232 and C_v_ = 0.300%, and L_Hu_ = 80.350 with S = 0.223 and C_v_ = 0.276% respectively (Table 1).

**Figure 5.**
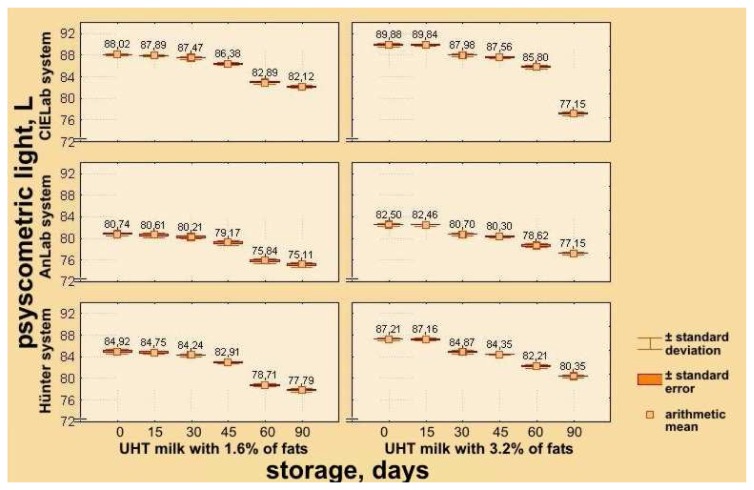
Effects of storage time on changes of psychometric lightness of UHT milk samples with 1.6% and 3.2% milk fat in CIELab, ANLAB and Hünter's systems.

Values for psychometric lightness for UHT milk samples with 1.6% milk fat are lower in all storage periods, if compared with the corresponding samples of milk with higher percentual milk fat (Table 2; Figure 5).

**Figure 6.**
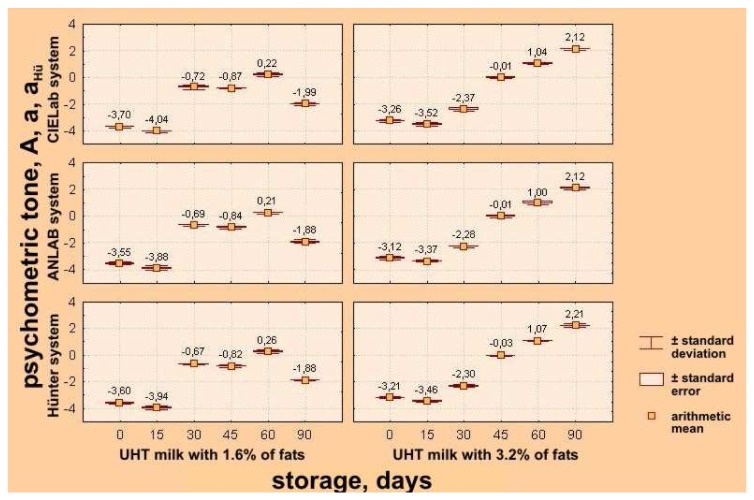
Effects of storage time on changes of psychometric tone of UHT milk samples with 1.6% and 3.2% milk fat in CIELab, ANLAB and Hünter's systems.

It is very important emphasize that values of measured color parameters such as psychometric tone (a*, A, a_Hu_) have a negative sign up to 60 days of storage for both groups of UHT milk samples, indicating the presence of components of a green (−) milk color; but, in period lasting from day 60 till day 90, the psychometric tone value was positive (+) (participation of red color components of milk color) (Table 2, Figure 6). According to Toba *et al.* [23], it is possible that getting darker (increasing of participation of red color) during degradation of tryptophan and tyrosine, can induce the mentioned color change of UHT milk during its exposure to light.

Calculated values for psychometric chroma (b*, B, b_Hu_) for UHT milk samples with 3.2% and 1.6% milk fat have a positive sign during the whole storage period, indicating the presence of components of yellow color of milk. Immediately after processing, the value for b* in the CIE L*a*b* system for UHT milk samples with 3.2% milk fat is b* = 9.270 with S = 0.373 and C_v_ = 4.026%. After 15 days of storage the b* value was increased insignificantly, reaching b* = 9.370 with S = 0.170; C_v_ = 1.819%, and after 30 days it was insignificantly lower with respect to the starting day, being b* = 9.370 with S = 0.170 and C_v_ = 1.819%; however, these changes were not statistically significant (p > 0.05). During further storage, i.e. after 45, 60 and 90 days, psychometric chroma values show a further decrease, so that on the day 90 we have: b* = 7.060 with S = 0.183 and C_v_ = 2.596% (Table 1). After 30 and 45 days the mean values of psychometric chroma (b*, B and b_Hu_) are statistically very significantly higher if compared with results obtained in the following measurements. From day 60 till to day 90 values of psychometric chroma decrease, but this was not statistically significant (p > 0.05).

**Figure 7.**
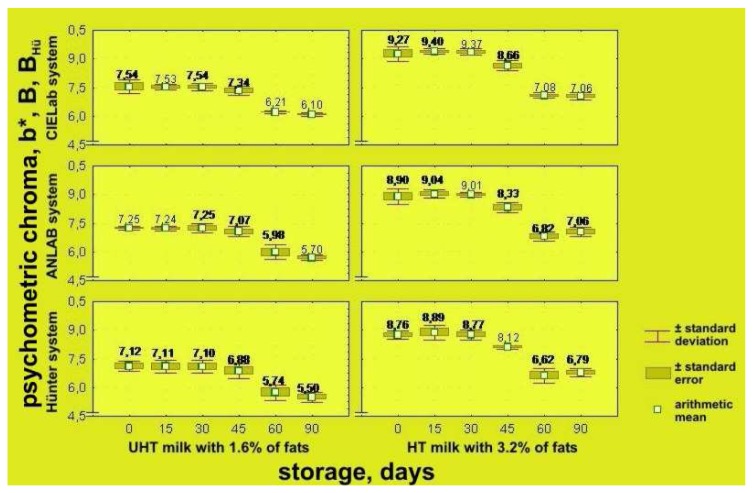
Effects of storage time on changes of psychometric chroma of UHT milk samples with 1.6% and 3.2% milk fat in CIELab, ANLAB and Hünter's systems.

The same regularity applies also for UHT milk samples with lower milk fat contents. So, immediately after processing, values for psychometric chroma are b* = 7.540 with S = 0.366 and C_v_=4.853%; on day 15 it was b* = 7.530 with S = 0.146 and C_v_ = 1.945%, and on day 30 it was identical with the value registered at the very beginning the of color analysis of the UHT milk with 1.6% milk fat. After day 90, the psychometric chroma value decreases (share of components of yellow color of milk) and reaches b* = 6.100 with S = 0.085 and C_v_=1.393% (Table 2, Figure 7). As a positive value of psychometric chroma b* indicates the participation of components of yellow color of milk, a continuous decrease of the b* value for UHT milk samples with 3.2 and 1.6% milk samples during storage (under conditions that simulate milk storage in practice), means a decrease of the share of yellow color. According to literature data [23-25], this change of color is probably induced by simultaneous degradation of the yellowish-green colored riboflavin (vitamin B_2_), β-carotene and vitamin A molecules.

On the basis of results obtained with MANOVA, the values of psychometric chroma (b*, B and b_Hu_) have changed statistically very significantly (p < 0.01) under the influences of the analyzed factors: defining system of color parameters (F = 30.539 and p=0.000), observed participation of milk fat (F = 817.570 and p = 0.000), as well as effect of storage time (F = 206.906 and p=0.000).

On the basis of the results of the LSD test for UHT milk with 1.6% milk fat, a statistically significant decrease (p < 0.05) of the average value of the psychometric chroma occurred on day 60 and for UHT milk with 3.2% milk fat on day 45. At each analysis point (days 0, 15, 30, 45, 60 and 90), the average psychometric chroma was statistically very significantly higher (p < 0.01) for milk with 3.2% milk fat.

Results of whiteness of UHT milk samples with 3.2% milk fat (ΔH_ab_; ΔE_AN_ and ΔE_Hu_) that were stored for 90 days (20 ± 5°C) deviated more from the basic model for white color, than results for whiteness of UHT milk samples containing 1.6% fat. Intensities of the observed differences for both groups of UHT milk samples increased with time of storage (Tables 1 and 2). Obtained results are in accordance with Cais-Sokolińska [5] asserting that calculated value of difference milk color (ΔE) is best to be applied as an intensity indicator of Maillard's reaction.

## 4. Conclusions

On the basis of obtained results, it is possible to state the following:

Average value of the mean reflectance, i.e. of brilliance of UHT milk with 3.2% and 1.6% milk fat statistically is changed very significantly (p < 0.01) under the influences of the analyzed factors: storage time (F = 1752.216 and p = 0.000) and milk fat content (F = 1428.497 and p = 0.000), as well as the influence of interactions of the mentioned factors (F = 47.590 and p = 0.000).

Values for psychometric chroma (b*, B and b_Hu_) are changed statistically very significantly (p < 0.01) depending on the system of definition of color quality parameters (F = 30.539 and p = 0.000), percentual share of milk fat (F = 817.570 and p = 0.000), as well as storage time (F = 206.906 and p = 0.000).

UHT milk samples with 1.6% milk fat showed statistically a very significant decrease (p < 0.05) of psychometric chroma after 60 days, and UHT milk samples with 3.2% milk fat after 45 days.

At each analysis point (days 0, 15, 30, 45, 60 and 90), the average psychometric chroma b* was statistically very significantly higher (p < 0.01) for samples of UHT milk with 3.2% milk fat, than for samples of UHT milk with 1.6% milk fat.

## Figures and Tables

**Table 1.** Results of instrumental determination of color of UHT milk with 3.2% milk fat during storage up to 90 days at ambient temperature (20 ± 5°C).

**Storage (days)**	**Statistical parameter**	**CIE system**			CIE L*a*b* system				**ANLAB system**				**Hünter's system**			
		**Calculated and read values**														
		**y (%)**	**λ(nm)**	**P (%)**	L*	a*	b*	**ΔH_ab_**	**L**	**A**	**B**	**ΔE_AN_**	**L_Hu_**	**a_Hu_**	**b_Hu_**	**ΔE_Hu_**
**0**	*X̅*	76.060	570	4.82	89.880	-3.260	9.270	2.840	82.500	-3.120	8.900	8.730	87.210	-3.210	8.760	8.860
	*S*	0.139	-	-	0.339	0.075	0.373	0.106	0.263	0.140	0.402	0.040	0.282	0.087	0.235	0.021
	*Cv*	0.183	-	-	0.377	8.694	4.026	3.728	0.318	14.001	4.517	0.463	0.323	9.531	2.679	0.235
**15**	*X̅*	75.970	570	4.82	89.840	-3.520	9.400	2.800	82.460	-3.370	9.040	8.920	87.160	-3.460	8.890	9.050
	*S*	0.274	-	-	0.204	0.137	0.134	0.047	0.180	0.075	0.214	0.142	0.238	0.093	0.384	0.126
	*Cv*	0.361	-	-	0.227	22.625	1.429	1.684	0.218	9.963	2.364	1.590	0.273	14.007	4.321	1.390
**30**	*X̅*	72.040	570	4.82	87.980	-2.370	9.370	3.140	80.700	-2.280	9.010	9.330	84.870	-2.300	8.770	9.760
	*S*	0.196	-	-	0.269	0.150	0.170	0.095	0.217	0.600	0.107	1.148	0.384	0.121	0.260	0.111
	*Cv*	0.272	-	-	0.306	8.580	1.819	3.024	0.267	3.270	1.187	1.590	0.450	6.622	2.967	1.133
**45**	*X̅*	71.160	570	4.82	87.560	-0.010	8.660	3.760	80.300	-0.010	8.330	8.950	84.350	-0.030	8.120	9.570
	*S*	0.297	-	-	0.204	0.083	0.250	0.059	0.190	0.095	0.281	0.045	0.127	0.035	0.100	0.091
	*Cv*	0.417	-	-	0.233	2.024	2.888	1.557	0.237	2.321	3.368	0.504	0.151	0.858	1.233	0.948
**60**	*X̅*	67.600	615	1.6	85.800	1.040	7.080	3.830	78.620	1.000	6.820	8.980	82.210	1.070	6.620	10.230
	*S*	0.359	-	-	0.391	0.087	0.142	0.070	0.358	0.159	0.231	0.047	0.292	0.055	0.367	0.178
	*Cv*	0.532	-	-	0.456	1.692	2.002	1.828	0.455	3.111	3.391	0.526	0.355	1.061	5.540	1.738
**90**	*X̅*	64.570	615	1.6	77.150	2.120	7.060	4.350	77.150	2.120	7.060	10.500	80.350	2.210	6.790	12.100
	*S*	0.296	-	-	0.394	0.074	0.183	0.091	0.232	0.101	0.251	0.067	0.223	0.112	0.235	0.095
	*Cv*	0.458	-	-	0.510	1.181	2.596	2.084	0.300	1.644	3.558	0.634	0.276	1.761	3.457	0.781

**Table 2.** Results of instrumental determination of color of UHT milk with 1.6% milk fat during storage up to 90 days at ambient temperature (20 ±5°C).

**Storage (days)**	**Statistical parameter**	**CIE system**			CIE L*a*b* system				**ANLAB system**				**Hünter's system**			
		**Calculated and read values**														
		**y (%)**	**λ(nm)**	**P (%)**	L*	a*	b*	**ΔH_ab_**	**L**	**A**	**B**	**ΔE_AN_**	**L_Hu_**	**a_Hu_**	**b_Hu_**	**ΔE_Hu_**
**0**	*X̅*	72.120	570	4.82	88.020	-3.700	7.540	2.330	80.740	-3.550	7.250	8.130	84.920	-3.600	7.120	8.600
	*S*	0.265	-	-	0.163	0.032	0.366	0.047	0.304	0.112	0.111	0.057	0.424	0.081	0.278	0.045
	*Cv*	0.367	-	-	0.185	7.593	4.853	2.025	0.376	19.600	1.536	0.699	0.499	15.560	3.905	0.524
**15**	*X̅*	71.840	570	4.82	87.890	-4.040	7.530	2.030	80.610	-3.880	7.240	8.190	84.750	-3.940	7.110	8.830
	*S*	0.442	-	-	0.138	0.072	0.146	0.064	0.461	0.154	0.116	0.084	0.345	0.150	0.341	0.117
	*Cv*	0.616	-	-	0.157	86.810	1.945	3.162	0.572	63.170	1.602	1.024	0.407	82.060	4.789	1.327
**30**	*X̅*	70.970	570	4.82	87.470	-0.720	7.540	3.240	80.210	-0.690	7.250	8.120	84.240	-0.670	7.100	8.790
	*S*	0.085	-	-	0.290	0.166	0.176	0.081	0.313	0.081	0.231	0.127	0.241	0.032	0.327	0.093
	*Cv*	0.120	-	-	0.332	4.884	2.337	2.492	0.391	2.352	3.181	1.559	0.286	0.930	4.611	1.057
**45**	*X̅*	68.750	570	4.82	86.380	-0.870	7.340	3.130	79.170	-0.840	7.070	8.500	82.910	-0.820	6.880	9.580
	*S*	0.244	-	-	0.267	0.006	0.225	0.006	0.459	0.122	0.248	0.096	0.175	0.101	0.387	0.096
	*Cv*	0.355	-	-	0.309	0.177	3.064	0.184	0.580	3.709	3.504	1.130	0.211	3.062	5.625	1.002
**60**	*X̅*	61.960	615	1.6	82.890	0.220	6.210	3.280	75.840	0.210	5.980	10.740	78.710	0.260	5.740	12.630
	*S*	0.218	-	-	0.311	0.139	0.066	0.075	0.438	0.055	0.386	0.035	0.236	0.138	0.416	0.140
	*Cv*	0.352	-	-	0.375	3.193	1.056	2.302	0.578	1.270	6.443	0.327	0.300	3.147	7.255	1.109
**90**	*X̅*	60.520	615	1.6	82.120	-1.990	6.100	3.150	75.110	-1.880	5.700	10.200	77.790	-1.880	5.500	11.870
	*S*	0.361	-	-	0.299	0.075	0.085	0.057	0.448	0.117	0.145	0.153	0.352	0.059	0.248	0.038
	*Cv*	0.596	-	-	0.364	3.545	1.393	1.803	0.596	5.224	2.540	1.500	0.453	2.612	4.517	0.319

## References

[1] Đorđević J. (1987). Mleko– fizika i hemija mleka..

[2] Niketiæ G., Maæej O., Jovanoviæ S. (2000). Uticaj termièkog tretmana na promene sastava UHT sterilizovanih proizvoda. Arhiv. Poljopr. Nauke..

[3] Jovanoviæ S., Maæej O., Jokiæ A., Mikuljanac A. (1997). Promena sadržaja laktoze u mleku u zavisnosti od promenjenih režima termièke obrade. Prehrambena industrija, Zbornik radova XII Savetovanja – Savremeni pravci razvoja u tehnologiji mleka..

[4] Maæej O., Jovanoviæ S., Denin–Đurđević J. (2002). Uticaj visokih temperatura na proteine mleka. Mlekarstvo.

[5] Cais-Sokolińska D., Pikul J., Danków R. (2004). Measurement of colour parameters as index of the hidroxymethylfurfural content in the UHT sterilised milk during its storage. Electron. J. Pol. Agric. Univ..

[6] Mestdagh F., De Meulenaer B., De Clippeleer J., Devlieghere F., Huyghebaert A. (2005). Protective Influence of Several Packing Materials on Light Oxidation of Milk. J. Dairy Sci..

[7] Rufián-Henares J.A., Guerra-Hernandez E., García-Villanova B. (2006). Colour measurement as indicator for controlling the manufacture and storage of enteral formulas. Food Control.

[8] Nielsen B. R., Stapelfeldt H., Skibsted L.H. (1997). Differentiation Between 15 Whole Milk Powders in Rotation in Relation to Oxidative Stability During Accelerated Storage: Analysis of Variance and Cannonical Variable Analysis. Int. Dairy J..

[9] Rizzi G.P. (1977). Chemical structure of coloured Maillard reaction products. Food Rev. Int..

[10] Ames J.M., Apryantano A., Arnoldi A. (1993). Low molecular weight colored compounds formed in xylose-lysine model systems. Food Chem..

[11] Ingles D. L., Gallimore D. (1985). A new method for the isolation of mellanoidins from the Maillard reaction of glucose and glycine. Chem. Ind. (London).

[12] Kessler H.G., Fink R. (1986). Changes in heated and stored milk an interpretation by reaction kinetics. J. Food Sci..

[13] Gaucher I., Mollè D., Gagnaire V., Gaucheron F. (2008). Effect of storage temperature on physicochemical characteristics of semi-skimmed UHT milk. Food Hydrocolloid..

[14] Kneifel W., Ulberth F., Schaffer E. (1992). Tristymulus colour reflectance measurement of milk and dairy products. Lait.

[15] Singh H., Creamer L.K., Fox P. F. (1992). Heat stability of milk. Advanced Dairy Chemistry Proteins, 1.

[16] Min D.B., Boff J.M. (2002). Chemistry and reaction of singlet oxigen in foods. Compr. Rev. Food. Sci. Food Safety.

[17] Borle F., Sieber R., Bosset J.O. (2001). Photo-oxidation and photoprotection of foods with particular reference to dairy products: An update of review article (1993-2000). Sci. Aliments.

[18] Bekbölet M. (1990). Light effects on foods. J. Food Prot..

[19] Saidi B., Warthesen J.J. (1995). Effect of heat and homogenization on riboflavin photolysis in milk. Int. Dairy J..

[20] Sears F.W. (1963). Optika..

[21] (1986). CIE Colorimetry Committee: Technical notes: working program on colour differences. J. Opt. Soc. Am..

[22] Robertson A.R. (1977). The CIE 1976 color difference formulae. Colour Res. Appl..

[23] Toba T., Adachi S., Arai J. (1980). Sunlight and sodium hypochlorite-induced colour changes in milk. J. Dairy Sci..

[24] Bosset J.O., Gallmann P.U., Sieber R. (1994). Influence of light transmittance of packaging materials on the shelf-life of milk and dairy products: A review. Food Packaging and Preservation.

[25] Lee K.H., Jung M.Y., Kim S.Y. (1998). Effects of ascorbic acid on the light-induced riboflavin degradation and colour changes in milk. J. Agric. Food Chem..

